# What Do Patients Think about the Cause of Their Mental Disorder? A Qualitative and Quantitative Analysis of Causal Beliefs of Mental Disorder in Inpatients in Psychosomatic Rehabilitation

**DOI:** 10.1371/journal.pone.0169387

**Published:** 2017-01-05

**Authors:** Julia Luise Magaard, Holger Schulz, Anna Levke Brütt

**Affiliations:** Department of Medical Psychology, Center for Psychosocial Medicine, University Medical Center Hamburg-Eppendorf, Hamburg, Germany; Chiba Daigaku, JAPAN

## Abstract

**Background:**

Patients’ causal beliefs about their mental disorders are important for treatment because they affect illness-related behaviours. However, there are few studies exploring patients’ causal beliefs about their mental disorder.

**Objectives:**

(a) To qualitatively explore patients’ causal beliefs of their mental disorder, (b) to explore frequencies of patients stating causal beliefs, and (c) to investigate differences of causal beliefs according to patients’ primary diagnoses.

**Method:**

Inpatients in psychosomatic rehabilitation were asked an open-ended question about their three most important causal beliefs about their mental illness. Answers were obtained from 678 patients, with primary diagnoses of depression (N = 341), adjustment disorder (N = 75), reaction to severe stress (N = 57) and anxiety disorders (N = 40). Two researchers developed a category system inductively and categorised the reported causal beliefs. Qualitative analysis has been supplemented by logistic regression analyses.

**Results:**

The causal beliefs were organized into twelve content-related categories. Causal beliefs referring to “problems at work” (47%) and “problems in social environment” (46%) were most frequently mentioned by patients with mental disorders. 35% of patients indicate causal beliefs related to “self/internal states”. Patients with depression and patients with anxiety disorders stated similar causal beliefs, whereas patients with reactions to severe stress and adjustment disorders stated different causal beliefs in comparison to patients with depression.

**Limitations:**

There was no opportunity for further exploration, because we analysed written documents.

**Conclusions:**

These results add a detailed insight to mentally ill patients’ causal beliefs to illness perception literature. Additionally, evidence about differences in frequencies of causal beliefs between different illness groups complement previous findings. For future research it is important to clarify the relation between patients’ causal beliefs and the chosen treatment.

## Introduction

Patients’ beliefs about their mental disorder are important for treatment because they affect illness-related behaviours [1, 2] and treatment outcomes like quality of life and psychological health [2–4]. For instance, those impacts were shown in patients with depression [1, 4], with psychotic disorders or personality disorder [2] and in a sample of patients with mixed mental disorders like depression, anxiety, stress-related and somatoform disorders [3]. The common sense model of illness representations by Leventhal, Nerenz and Steele [5] comprises a theoretical basis for patients’ beliefs and their impact on illness related behaviours. Leventhal and colleagues [5] proposed that patients’ perceptions about their illness are based around five distinct components, namely identity, timeline, cause, consequences and cure/control. The model states that these perceptions affect patients’ coping behaviour and their appraisal of the outcome of their efforts. This model is often used and empirically confirmed in patients with somatic illnesses [6].

### Causal Beliefs of Patients with Mental Disorders

Patients’ causal beliefs of their mental illnesses represent an essential component of these illness perceptions. Studies showed that mentally ill patients’ causal beliefs are associated with impairment [7, 8], coping [1, 2, 9, 10] and outcome [8, 10, 11]: For instance, Cornwall, Scott (7) concluded that biological causal beliefs of patients with depression were associated with severity of depression. Broadbent, Kydd (2) reported that patients with psychotic or personality disorders who believed in psychosocial primary causes were more likely to attend their GP frequently than those who believed in behavioural causes [2]. Investigating patients with major depression in a clinical trial of alternative medicine (hypericum perforatum), Bann and colleagues [8] showed that strong external causal beliefs, i.e. biological abnormality, were associated with less improvement regarding symptoms. Consequently, the exploration of patients’ causal beliefs is relevant in research and clinical practice.

Although considerable research about patients’ causal beliefs of mental illnesses is available, there is little consensus on which causal beliefs are most important to which groups of patients (e.g. [7, 8, 10, 11, 12, 13–18]). Mentally ill patients’ causal beliefs were most commonly measured through questionnaires and patients rate each cause from a giving list as regards to how much they agree with it (e.g. [7, 8, 10, 11, 14, 19, 20, 21]). In these studies, patients diagnosed with mental disorders agreed with a variety of causal beliefs including biological (e.g. [8, 11, 20, 21]), psychological (e.g. [8, 19, 20, 21]), stress related (e.g. [11, 14, 20, 21]) and social factors (e.g. [19, 20, 21]). Using these questionnaires, the degree of agreement can be assessed, but a subjective estimate of the relevance of the causal beliefs is not possible. Qualitative approaches enable gaining a deeper insight into patients’ causal beliefs by directly asking patients about their own ideas in their own words and focusing on relevant causal beliefs from patients’ perspectives. For instance, qualitative studies explored causal beliefs of mentally ill in-patients [17], patients from a community mental health centre [16] diagnosed with different disorders (e.g. depression, anxiety, bipolar disorder, borderline personality disorder, obsessive compulsive disorder, schizophrenia, alcohol abuse) and primary care patients with depression [12]. Causal beliefs like life experiences, social or environmental situations, physical or biological factors [16, 17] as well as personal mistakes [17] were identified. Hansson et al. [12] asked primary care patients with depression an open-ended question about what had caused their depression and organized their data into three larger themes: “current life stressors” covered problems related to work, family, somatic illnesses, loneliness and finances; “past life events” referred to experiences in the past and “constitution” covered aspects of personality and disposition.

### Differences in Causal Beliefs

Some studies investigated differences in causal belief among patients with depression according to clinical and sociodemographic variables [11, 12, 22]. For instance, the length of time taking antidepressants was positively correlated with bio-genetic beliefs in a New Zealand sample [11]. Hansson et al. [12] found that women with depression reported family related problems and past life events like childhood to a higher extent than man. Work related stress was the most frequently mentioned causal belief for depression in all age groups, but less likely mentioned by adults aged 60 to 69 years [12]. Causal beliefs referring to childhood and personality were more frequently mentioned by young adults aged 18 to 39 years [12]. Middle aged (40–59) attributed their depression more often to life events like separation whereas older adults mentioned causal beliefs like death of a friend or relative more frequently [12]. Assessing causal beliefs about depression among Latino immigrants in primary care Caplan, Paris (22) identified that depression, age, country of origin and religiosity were significantly associated with causal beliefs. According to gender no differences were identified in this study [22]. Results of studies investigating illness-specific causal beliefs among different groups of patients (e.g. depression, psychosis) are available and studies exploring samples of patients with different mental disorders have been published. So far, studies comparing these beliefs between patients with different diagnoses are lacking.

Based on the existing literature, a supplementary precise analysis of causal beliefs in patients with mental disorder supplemented by analyses of differences between different mental disorders is reasonable.

In order to explore mentally ill patients’ causal beliefs, the first aim of this study was to qualitatively explore and categorise patients’ beliefs about the cause of their mental disorder. The second aim of the study was to explore frequencies of patients stating at least one causal belief assigned to a category. The third aim of the study was to compare causal beliefs mentioned by patients with a primary diagnosis of depression to causal beliefs mentioned by patients with primary diagnoses of adjustment disorder, reaction to severe stress and anxiety disorder controlling for age, gender, psychopharmacologic drug use and employment status.

## Materials and Methods

### Procedure

A consecutive sample of n = 712 patients with mental disorders was recruited from four cooperating rehabilitation centres between January 2013 and September 2013. Patients were undergoing inpatient treatment as part of psychosomatic rehabilitation, focusing on the reduction of symptoms and the improvement of social and work participation. The basic treatment included single and group psychotherapy, psychopharmacotherapy as well as psychoeducation. Additionally, the integrated rehabilitative approach also offered physical therapy or training in coping skills [23]. All participants provided written informed consent. The study was approved by the local Ethics Committee of the Hamburg Medical Chamber (reference number: PV4207) and was conducted according to the principles expressed in the Declaration of Helsinki.

### Data Collection

Participants filled in a questionnaire about demographic information, use of psychopharmaceuticals, patients’ risks and resources, functioning and psychosocial health at admission. As a part of the questionnaire, they were asked to answer an open-ended question about their three major beliefs about what had caused their mental illness. The item “Please list in rank-order the three most important factors that you believe caused your mental disorder.” was adapted from the revised illness perception questionnaire (IPQ) [24]. Primary diagnoses according to ICD-10 [25] criteria were assessed by treatment providers at admission of psychosomatic rehabilitation.

### Participants

Of 712 recruited patients, 678 patients stated at least one reason for their mental disorder. The following characteristics describe this sample: Most of the patients were women (73%), their mean age was 47 years (SD = 12) and N = 341 had a primary diagnosis of depression (depressive episode (F32) or recurrent depressive disorder (F33) currently mild, moderate, severe without psychotic symptoms or not otherwise specified) according to the ICD-10 criteria [25]. Further primary diagnoses were adjustment disorder (N = 75), reaction to severe stress (PTSD or acute stress reaction; N = 57), and anxiety disorders (N = 40). The sample characteristics represent patients’ characteristics in psychosomatic inpatient rehabilitation at admission [23]. Item-responders and non-responders did not differ regarding age (p = .164), gender (χ^2^(1,694) = 2.346, p = .126) and primary diagnoses (χ^2^(10,620) = 5.112, p = .884).

Table 1 shows the patient characteristics of the total.

**Table 1:** Sample characteristics.

Characteristics		Total sample
N		678
Age (M, SD)		46.77 (11.78)
Gender (N, %)		
	Women	481 (72.8)
	Man	180 (27.2)
Length of rehabilitation in weeks (M, SD)		5.10 (1.27)
Level of Education (N, %)		
	Elementary school	170 (26.0)
	Secondary school/technical Vocational school	269 (41.2)
	University qualification	177 (27.1)
	Other	37 (5.7)
Married (N, %)		
	Yes	307 (47.9)
	No	334 (52.1)
Employment status (N, %)		
	Employed	406 (61.5)
	Not employed	254 (38.5)
Citizenship (N, %)		
	German	641 (97.1)
	Other	19 (2.9)
Diagnostic group (N, %)		
	Depressive disorder	341 (57.8)
	Adjustment disorders	75 (12.7)
	Reaction to severe stress	57 (9.7)
	Anxiety disorders	40 (6.8)
	Somatoform disorders	31 (5.3)
	Other mental disorders	42 (7.7)
Psychopharmacologic drug use (N, %)		
	Psychopharmacological drug use	331 (51.2)

### Data Analyses

#### Qualitative data analysis

According to thematic analysis principles [26] two researchers (ALB and JLM) defined categories inductively. First, the patients’ answers were read through several times. Both researchers separately aggregated similar causal statements into categories. Different levels of themes, categories and subcategories were identified. These categories were discussed to develop a harmonized category system including category definitions. Afterwards, the two researchers assigned all statements to the category system. An inter-rater reliability of Kappa = .82 was accomplished on the level of subcategories in the categorization process. Mismatching categorizations were discussed until consensus was reached (JLM, ALB). Additionally, feedback from external reviewers was used to further specify the category system. As a result, an overview of categories and subcategories of patients’ causal beliefs was developed. The qualitative analysis was performed with MAXQDA 10.

#### Quantitative data analysis

All statistical analyses were performed with SPSS 18. Frequencies of coded categories were calculated and reported in the category system on the level of responses. In addition, frequencies were reported on patients-level as percentages of patients who mentioned at least one causal belief referring to the different categories.

Finally, multivariate logistic regressions were conducted to estimate the probability of a dichotomous response to each causal belief (stated vs. not stated) on the nominal predictor variable primary diagnoses (depression, adjustment disorder, reaction to severe stress, anxiety disorder). To adjust regression models for effects of covariates, age, gender, psychopharmacologic drug use and employment status were included in step 1. In step 2 the nominal predictor primary diagnoses was added. If variance of a predictor regarding a causal belief was lacking, analyses were not conducted for that causal belief [27]. Consequently, seven logistic regressions estimating the probability of the response to seven causal beliefs were conducted. Results are presented as adjusted odds ratios (AORs) with 95% confidence intervals (CIs). The coefficient of determination Nagelkerkes’ Pseudo-R^2^ [28] ranging between 0 and 1 was calculated to interpret the proportions of explained variation in the dichotomous response to each causal belief (stated vs. not stated). Nagelkerkes’ Pseudo-R^2^ > 0.2 are considered acceptable [29].

## Results

### Causal Beliefs of Patients with Mental Disorders

#### Category system of causal beliefs

In total, 1858 reasons were stated and assigned to the category system. Each item-responding patient named 2.74 (SD = 0.58) causal beliefs on average.

**Fig 1:**
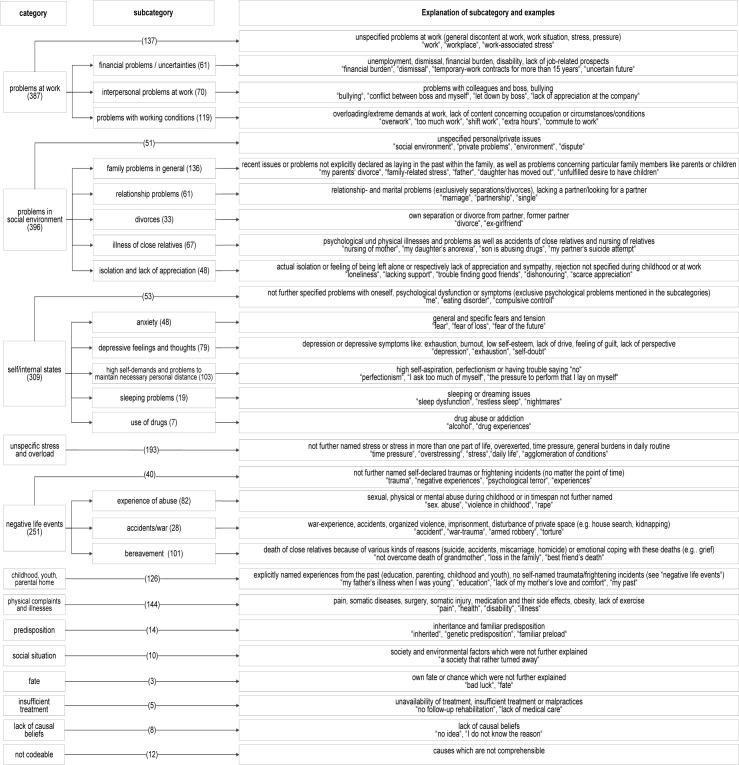
Category system of causal beliefs of mental disorders. Frequencies of coded categories were reported for each category on the level of responses (N = 1858).

The causal beliefs were organized into twelve content-related categories. Fig 1 displays the categories, subcategories, explanations of categories and subcategories and examples of the patients’ beliefs about the cause of their mental disorder. The categories “problems at work”, “problems in social environment”, “self/internal states” and “negative life events” can be differentiated in subcategories.

In summary, most of the patients (45%) mentioned causal beliefs out of two different categories. Moreover, 37% of the patients mentioned causal beliefs referring to three categories, whereas 18% of the patients mentioned causal beliefs referring to only one category. Two patients reported a lack of causal beliefs and one patient reported only incomprehensible causal beliefs. “Problems in social environment” and “problems at work” were often mentioned together.

#### Frequencies of causal beliefs of patients with mental disorders

47% of the patients diagnosed with a mental disorder mentioned at least one cause out of the category “problems at work” and nearly the same percentage of patients mentioned at least one cause out of the category “problems in social environment” (46%). Additionally, more than one third (35%) indicated at least one cause referring “self/internal states”. Causal beliefs assigned to the category “negative life events” were mentioned by 29% of the patients and 24% of the patients indicated at least one cause referring to “unspecific stress and overload”. At least one cause out of the category “physical complaints and illnesses” or “childhood, youth, parental home” were stated by 17% of the patients. “Predisposition”, “social situation”, “insufficient treatment”, “fate” and “lack of causal beliefs” were stated very rarely. Some patients (2%) mentioned at least one cause that could not be coded. Table 2 shows the frequencies of causal beliefs stated by patients with mental illnesses.

**Table 2:** Percentage of patients stating at least one causal belief in that category (n = 678).

Category	Subcategory	%^a^	N
**Problems at work**		**46.5**	**315**
	Problems with working conditions	15.5	105
	Interpersonal problems at work	10.0	68
	Financial problems / uncertainties	8.6	58
	Unspecified	19.5	132
**Problems in social environment**		**46.2**	**313**
	General family problems	19.2	130
	Illness of close relatives	8.7	59
	Relationship problems	8.6	58
	Isolation and lack of appreciation	6.6	45
	Divorces	4.4	30
	Unspecified	6.8	46
**Self/internal states**		**34.8**	**236**
	High demands on themselves and problems to maintain the necessary personal distance	13.3	90
	Depressive feelings and thoughts	10.5	71
	Anxiety	6.8	46
	Sleeping problems	2.8	19
	Use of drugs	1.0	7
	Unspecified	7.7	52
**Unspecific stress and overload**		**23.6**	**160**
**Negative life events**		**28.5**	**193**
	Bereavement	13.7	93
	Experience of abuse	9.4	64
	Accidents/war	3.7	25
	Unspecified	5.9	40
**Childhood, youth, parental home**		**17.4**	**118**
**Physical complaints and illnesses**		**17.4**	**118**
**Predisposition**		**2.1**	**14**
**Social situation**		**1.3**	**9**
**Insufficient treatment**		**0.7**	**5**
**Fate**		**0.3**	**2**
**Lack of causal beliefs**		**1.2**	**8**
**Not codeable**		**1.6**	**11**

^a^Percentage of patients, who mentioned at least one cause referring to this category

#### Comparison of causal beliefs about depression with causal beliefs about other diagnoses

Multivariate logistic regressions were conducted to examine whether patients’ primary diagnoses predicted presence of specific casual beliefs, adjusting for age, gender, psychopharmacologic drug use and employment status (Table 3 and Table 4). The type of primary diagnoses was significantly associated with causal beliefs like “problems at work”, “problems in social environment”, “unspecific stress and overload”, “negative life events” and “childhood, youth, parental home”. Compared to the primary diagnosis of depression, the primary diagnosis of adjustment disorder was associated with increased likelihood of stating “negative life events” (adjusted odds ratio [AOR] = 2.05, 95% CI = 1.12–3.78) and with decreased likelihood of causal beliefs referring to “problems in social environment” (AOR = 0.53, 95% CI = 0.30–0.92) and “childhood, youth, parental home” (AOR = 0.19, 95% CI = 0.06–0.64). Also, compared to the primary diagnosis of depression, a primary diagnosis of reaction to severe stress was associated with increased likelihood of stating causal beliefs regarding “negative life events” (AOR = 11.77; 95% CI = 5.60–24.73) and with decreased likelihood of stating causal beliefs regarding “problems at work” (AOR = 0.23, 95% CI = 0.11–0.51) and “unspecific stress and overload” (AOR = 0.29, 95% CI = 0.10–0.83). Regarding potential confounders, gender and psychopharmacologic drug use were not significantly associated with all quantitatively investigated causal beliefs. However, adjusting for primary diagnoses, age was significantly related to “problems at work” (AOR = 1.02, 95% CI = 1.01–1.04), “problems in social environment” (AOR = 0.98, 95% CI = 0.96–0.99) and “physical complaints and illnesses” (AOR = 1.03, 95% CI = 1.01–1.06). Employment status was significantly associated with “problems at work” (AOR = 0.48, 95% CI = 0.32–0.72), “negative life events” (AOR = 1.78, 95% CI = 1.13–2.82) and “childhood, youth, parental home” (AOR = 1.81, 95% CI = 1.11–2.94), adjusting for primary diagnoses. Considering Pseudo-R^2^ (Nagelkerke) [28], the predictor variables primary diagnoses, age, gender, employment status and psychopharmacologic drug use explained between 2% (self/internal states) and 20% (negative life events) of the variation in the dichotomous response to each causal belief.

**Table 3:** Causal beliefs: logistic regressions, Odds ratio (Exp(b)).

		problems at work		problems in social environment		Self/internal states		Unspecific stress and overload	
		Step 1	Step 2	Step 1	Step 2	Step 1	Step 2	Step 1	Step 2
Age		1.02**	1.02*	0.98**	0.98**	0.99	0.99	1.00	1.00
Gender (reference: women)		1.52	1.48	0.78	0.83	1.14	1.10	0.63	0.60
Psychopharmacologic drug use (reference: no)		1.42	1.42	0.82	0.79	1.14	1.12	0.89	0.87
Employment status (reference: yes)		0.45**	0.48**	0.90	0.88	0.68	0.69	0.69	0.74
Primary diagnoses (reference: Depressive disorder)									
	Adjustment disorder		0.71		0.53*		0.96		0.82
	Reaction to severe stress		0.23**		0.73		0.75		0.29*
	Anxiety disorder		0.70		0.53		1.32		1.04
Constant (b)		0.33*	0.40*	3.32**	3.91**	0.85	0.85	0.35*	0.39
Pseudo-R^2^ (Nagelkerke)		0.09	0.13	0.03	0.05	0.01	0.02	0.02	0.04

N = 464.

*p < .05.

**p < .01.

**Table 4:** Causal beliefs: logistic regressions, Odds ratio (Exp(b)).

		Negative life events		Childhood, youth, parental home		Physical complaints and illnesses	
		Step 1	Step 2	Step 1	Step 2	Step 1	Step 2
Age		0.99	0.99	1.00	1.00	1.03*	1.03*
Gender (reference: women)		0.91	1.11	0.89	0.90	1.23	1.07
Psychopharmacologic drug use (reference: no)		1.14	1.26	1.57	1.39	0.89	0.88
Employment status (reference: yes)		2.00**	1.78*	1.90**	1.81*	1.05	1.20
Primary diagnoses (reference: Depressive disorder)							
	Adjustment disorder		2.05*		0.19**		1.42
	Reaction to severe stress		11.77**		0.98		0.15
	Anxiety disorder		0.78		1.11		2.19
Constant (b)		0.48	0.36*	0.16**	0.16**	0.03**	0.03**
Pseudo-R^2^ (Nagelkerke)		0.05	0.20	0.04	0.08	0.03	0.07

N = 464.

*p < .05.

**p < .01.

## Discussion

This study aimed to qualitatively explore patients’ causal beliefs about their mental disorders, to identify common causal beliefs and to investigate differences regarding causal beliefs between patients with different primary diagnoses. Therefore, the qualitative analysis of the three most important causal beliefs of psychosomatic inpatients was supplemented by logistic regression analyses regarding causal beliefs.

Results from our qualitative approach of asking open-ended questions about the most important causal beliefs complemented results from studies using questionnaires (e.g. [7, 8, 10, 11, 14, 19, 20, 21]), because attention was payed to the causal beliefs most important from the patients’ perspective.

Our qualitative approach resulted in a detailed insight to mentally ill patients’ causal beliefs. We described thirteen major categories with zero to seven subcategories per category. In line with other qualitative studies (e.g. [12, 16, 17]), our findings suggest that mentally ill patients hold a variety of different causal beliefs. Patients emphasized the etiological importance of current life stressors such as at work and in social environment, unspecific stress as well as previous life experiences such as negative life events and circumstances during childhood. Besides problems concerning the person itself, physical complaints and predisposition, fate and insufficient treatment were considered as causal for their mental disorders. This study added insufficient treatment to the already described causal beliefs from the current literature (e.g. [12, 16, 17]). Moreover, our analysis revealed new aspects to broader themes like interpersonal problems at work, illness of close relatives, high self-demands, sleeping problems, accidents and wars were derived from the material of the current study.

Analysing the frequencies of the three most important causal beliefs in the current study, it appeared that a relevant proportion of patients with mental disorders stated causal beliefs related to work (47%), social environment (46%), the self (35%), negative life events (29%), stress (24%), the childhood (17%) and physical complaints (17%). Although 49% of patients in our sample were on psychopharmacological medication, only a few stated biological factors like predisposition (2%) as one of the major three causal beliefs for their mental disorder. Using qualitative methods, similar findings were observed among patients with depression [12, 13] and patients with psychosis [10]. Using checklists contrasting results were found. For instance, Read et al. [11] reported that 85% of patients agreed with the statement that depression results from a chemical imbalance, 77% of patients agreed with depression due to heredity and 71% of patients regarded depression as a disorder of the brain. It is important to note that high percentages of participants also agreed on social causal beliefs like e.g. family stress (91%) and financial problems (87%) [11]. Additionally, in both studies conducted by Brown and colleagues [1, 9] 40% agreed with depression due to heredity. These findings support the view that there is a difference in answering open-ended questions and agreeing or disagreeing with statements. Hence, bio-medical explanations of mental disorders may be plausible for patients, but other causal beliefs seem to be most relevant. Another explanation for differing results may be cross-cultural differences in causal beliefs. Therefore, internationally conducted studies can help to clarify these differences.

Our study provides new insights into the differences between patients with different mental disorders regarding their causal beliefs about depression. Compared to patients with depression, patients with adjustment disorders or reaction to severe stress mentioned causal beliefs referring to negative life events more frequently. The results are in line with the scientific views on aetiology of these disorders, because they are triggered by a stressful or traumatic event [25]. Patients with depression had an increased likelihood to perceive causal beliefs referring to problems at work and unspecific stress compared to patients with reaction to severe stress. In addition, patients with depression also had an increased likelihood to consider aspects of social environment and childhood, youth and parental home as causal compared to patients with adjustment disorders. Patients with anxiety disorders did not differ from patients with depression regarding frequently stated causal beliefs. In our analyses employment status as well as age were significantly related to causal beliefs, whereas gender and psychopharmacologic drug use were unrelated in the adjusted models. For example, it makes sense, that age was positively related to the likelihood of stating causal beliefs regarding physical complaints and illnesses, because the likelihood of physical problems is raising with advancing age. Primary diagnoses, age, gender, employment status and psychopharmacologic drug use helped to explain different proportions of explained variation in stating each causal belief ranging between 2 and 20%. Further research is needed to prove or disprove these results.

### Implications for Future Research and Clinical Practice

As a result we identified categories of causal beliefs, which are adequate for different mental disorders. The category system creates an opportunity to systematically analyse the open-ended causal beliefs questions from the revised IPQ [24] or Brief-IPQ [30] in future research among patients with mental disorders. Further research is needed to examine if these categories match the causal beliefs of patients with psychiatric disorders like schizophrenia and patients with mental disorders due to psychoactive substance use. In clinical settings, multiple diagnoses are prevalent and therefore, illness causal perceptions may often not be considered separately. For future research it might be advisable to identify additional characteristics explaining more variation in stating different causal beliefs. Findings could help treatment providers to understand their patients’ causal beliefs and also improve shared-decision making on choosing the appropriate treatment. Knowledge about differences and similarities between patients with different mental disorders regarding their causal beliefs could contribute to individualize therapy.

### Study Limitations and Strengths

The strength of the current study includes that it provided insight into causal beliefs of patients with mental disorders by enriching a detailed qualitative analysis with multivariate logistic regression analyses. Compared to qualitative studies, a large sample of patients were surveyed in the current study. Regarding the quality of the qualitative analyses, the two researchers (ALB, JLM) which coded the causal beliefs were blinded for primary diagnoses of the patients. Written open-ended questions could have limited the risk of social desirability. However, because of written material it was not possible to clarify the statements of participants, especially in relation to the lifespan. Because of incomprehensibility twelve out of 1858 statements could not be categorised. Data collection was included in a larger study and patients filled in further questionnaires. However, we do not think that this influenced the answers to the causal beliefs questions, because priming effects of questions about demographic information, use of psychopharmaceuticals and patients’ risks and resources on causal beliefs were not expected. A non-responder analysis was only possible for item-non-responders. No information was available about non-responding participants. We assessed causal beliefs at the beginning of psychosomatic inpatient rehabilitation. It is important to replicate these results in different treatment settings, because it is possible, that seeking information in preparation for multicomponent treatment as offered in inpatient rehabilitation have already altered some causal beliefs. Especially causal beliefs of illness have been a predictor for treatment preference [31] and therefore our sample is prone to selection bias.

### Conclusion

This study showed that psychosomatic inpatients state a diversity of causal beliefs for their mental disorders and provided a category system to categorise these beliefs. Causal beliefs related to work, social environment, self, stress, negative life events, childhood and physical complaints were most prevalent among those patients, whereas only a few patients believed in causes like predisposition, social situation, fate or insufficient treatment. Patients with adjustment disorders or reaction to severe stress differed from patients with depression in terms of causal beliefs. Compared to patients with depression, patients with anxiety disorders had similar causal beliefs. The findings from the current study could help improve communication between treatment providers and patients and could help to individualize therapy.

## Abbreviations

IPQ: Illness perception questionnaire
AOR: adjusted odds ratios
CI: confidence intervalls
